# Phage‐mediated horizontal transfer of *Salmonella enterica* virulence genes with regulatory feedback from the host

**DOI:** 10.1002/imt2.70042

**Published:** 2025-05-20

**Authors:** Tianjing She, Demeng Tan, Jose Luis Balcazar, Ville‐Petri Friman, Danrui Wang, Dong Zhu, Mao Ye, Mingming Sun, Shujian Yuan, Feng Hu

**Affiliations:** ^1^ Soil Ecology Lab, Jiangsu Collaborative Innovation Center for Solid Organic Waste Resource Utilization and Jiangsu Key Laboratory for Solid Organic Waste Utilization Nanjing Agricultural University Nanjing China; ^2^ Shanghai Public Health Clinical Center Fudan University Shanghai China; ^3^ Catalan Institute for Water Research ICRA Girona Spain; ^4^ Department of Microbiology University of Helsinki Helsinki Finland; ^5^ Zhejiang Key Laboratory of Urban Environmental Processes and Pollution Control, Ningbo Urban Environment Observation and Research Station Chinese Academy of Sciences Ningbo China; ^6^ National Engineering Research Center for Soil Nutrient Management and Pollution Remediation, Institute of Soil Science Chinese Academy of Sciences Nanjing China

**Keywords:** horizontal gene transfer, phages, regulators, *Salmonella enterica*, virulence genes

## Abstract

Phage‐mediated horizontal transfer of virulence genes can enhance the transmission and pathogenicity of *Salmonella enterica* (*S. enterica*), a process potentially regulated by its regulatory mechanisms. In this study, we explored the global dynamics of phage‐mediated horizontal transfer in *S. enterica* and investigated the role of its regulatory mechanisms in transduction. A total of 5178 viral sequences encoding 12 *S. enterica* virulence genes were retrieved from the Integrated Microbial Genomes and Virome (IMG/VR) database, alongside 466,136 *S. enterica* genomes from EnteroBase. Virulence genes, including *iacP* (acyl carrier protein), *mgtB* (P‐type Mg^2+^ transporter), *misL* (autotransporter porin), and *fliC* (flagellar filament protein), were widely distributed in phages and *S. enterica* across North America, Europe, and Asia. Phylogenetic analysis revealed close genetic affinity between phage‐ and bacterial‐encoded virulence genes, suggesting shared ancestry and historical horizontal gene transfer events. The global regulator carbon storage regulator A (*csrA*) was highly conserved and ubiquitous in *S. enterica*. Overexpression of *csrA* inhibited prophage cyclization and release by upregulating the prophage *cI* repressor during horizontal gene transfer. Overall, these findings enhance our understanding of phage‐mediated horizontal transfer of virulence genes, explore new areas of bacterial regulators that inhibit gene exchange and evolution by affecting phage life cycles, and offer a novel approach to controlling the transmission of phage‐mediated *S. enterica* virulence genes.

## Highlights

- Phages carrying *Salmonella enterica* virulence genes are globally distributed across multiple habitats.
- Horizontal transfer of virulence genes occurs between phages and *Salmonella enterica*.
- The global regulator *csr*A is ubiquitous and genetically conserved in *Salmonella enterica*.
- *csr*A limits horizontal transfer of *Salmonella* virulence genes by inhibiting prophage cyclization and release in *Salmonella enterica*.

## Graphical abstract

In this study, we found evidence of phage‐mediated horizontal transfer of virulence genes in *Salmonella enterica* in global habitats from a molecular evolutionary perspective. Notably, *csr*A, a global regulator in *Salmonella enterica*, curbed horizontal gene transfer by inhibiting the cyclization and release of prophage. This is a novel finding in phage–bacteria interactions that provides new ideas for controlling pathogenic bacteria in the environment.

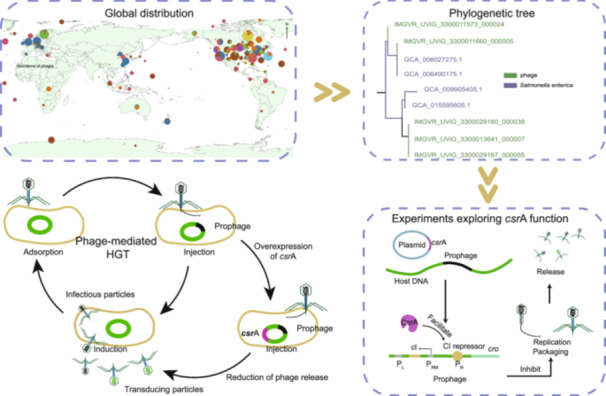

## INTRODUCTION

Infections caused by *Salmonella enterica* (*S. enterica*) can lead to typhoid and paratyphoid fevers, diarrheal illness, and, less commonly, blood infection. As a major cause of foodborne illness, *S. enterica* infections emerged from industrialized poultry farming in the 1980s in North America, South America, and Europe, and subsequently spread to various habitats around the world [1]. Decades after the initial salmonellosis outbreak, an estimated 17.8 million cases of typhoid fever caused by the pathogen occur annually in low‐ and middle‐income countries, making these infections an ongoing global health issue [2, 3]. Centralized sourcing and international trade of poultry breeding have primarily contributed to the global spread of *Salmonella* Enteritidis [1]. However, the global distribution pattern of *S. enterica* across different habitats remains largely unexplored.

The presence of virulence factors is an important prerequisite for pathogenicity, meaning that the outcome of *S. enterica* infection largely depends on whether the host bacterium carries these virulence factors. “Classical” virulence factors of *S. enterica* include virulence plasmids, toxins, pili, and flagella. For instance, the P‐type Mg^2+^ transporter encoded by *mgt*B helps *S. enterica* survive in phagocytes [4, 5], whereas the flagellar protein encoded by *fli*C is associated with bacterial motility [6, 7]. In addition, the autotransporter family porin encoded by *mis*L and the acyl carrier protein encoded by *iac*P play roles in bacterial colonization and invasion, respectively [8, 9]. Horizontal gene transfer (HGT) is a process in which genetic material is transferred between different organisms, rather than being passed down vertically from parent to offspring. Phage‐mediated HGT involves the packaging of DNA fragments from host bacteria into phage particles, followed by the transfer of these genes to other bacteria during infection. Phage‐mediated horizontal transfer of virulence genes is a crucial mechanism through which *S. enterica* acquires pathogenicity and adapts to ever‐changing environments [10, 11, 12]. For example, prophages can confer virulence to *Corynebacterium diphtheriae* and *Corynebacterium ulcerans* after lysogenization [13]. In *S. enterica*, phage P22 undergoes lateral transduction (LT), which facilitates high‐frequency transfer of host DNA and contributes to host evolution [14]. Patterns of virulence genes located close to phage integration sites (*att*B) and in the direction of packaging suggest that their transfer may be facilitated via LT [15]. However, there is currently no direct evidence of phage‐mediated horizontal transfer of *S. enterica* virulence genes.

Pathogenic bacteria, including *S. enterica*, encode a variety of regulators to modulate the expression of virulence genes. Major regulators include the carbon storage regulator A (*csr*A) that regulates *S. enterica* flagella expression, biofilm formation, *Salmonella* Pathogenicity Island 1 (SPI‐1) virulence genes, and carbon‐related genes [16, 17, 18, 19]; two‐component system BarA/SirA that affects SPI‐1 gene expression [20]; the histone‐like nucleoid structuring protein (H‐NS) that silences the expression of exogenous genes [21]; and the CRISPR‐Cas system that plays an important role in blocking the acquisition of exogenous genetic material and defending against phages [22]. As a result, these regulators are likely to influence the transmission of virulence genes, either promoting or repressing the expression of *S. enterica* virulence genes upon entry into host cells [23, 24]. However, the role of these regulators in HGT has not yet been explored, which is a crucial step in understanding the complex influence of phages on the horizontal transfer of virulence genes and in identifying potential strategies for its control.

In this study, we hypothesize that *S. enterica* virulence genes can be encoded and transferred by phages, which are further regulated by *S. enterica* regulatory genes. We obtained *S. enterica* genomes from Warwick's EnteroBase and phage genomes encoding *S. enterica* virulence genes such as *fli*C, *iac*P, *mgt*B, and *mis*L, among others, from the Integrated Microbial Genomes and Virome (IMG/VR) database (version 4). We investigated the geographical distribution of virulence genes in *S. enterica* and phages, analyzed the functional mechanisms and phylogenetic relationships of virulence factors and regulators, and experimentally assessed the effect of the *S. enterica* regulator *csr*A on transduction. The results may provide new scientific insights into the global scope of phage‐mediated horizontal transfer of virulence genes and offer strong support for strategies aimed at controlling the horizontal transfer of *S. enterica* virulence genes.

## RESULTS

### Geographic distribution of *S. enterica* virulence genes carrying bacteria and phages

**FIGURE 1:**
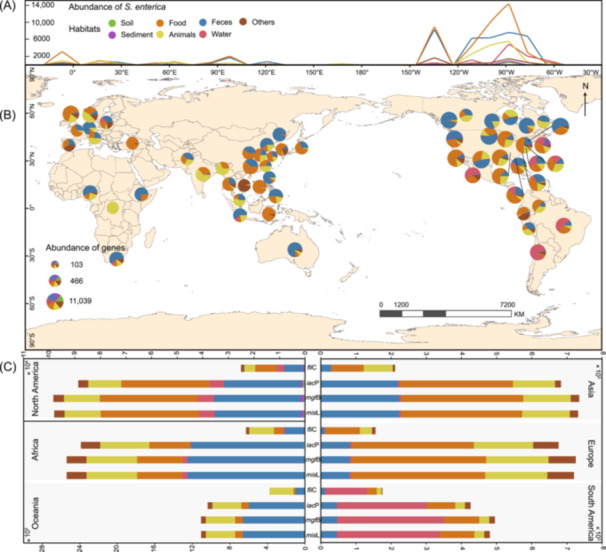
Global geographical distribution of *S. enterica*. (A) Patterns of global variation in *S. enterica* abundance with longitude. The *x*‐axis represents longitude and corresponds exactly to the longitude of the map. The *y*‐axis represents the abundance of *Salmonella* carrying virulence genes. The colors represent habitats. (B) Global distribution of *S. enterica* in different habitats. The size of the pie chart represents the abundance of *S. enterica*. And the colors represent habitats. (C) The abundance of four virulence genes (*fli*C, *iac*P, *mgt*B, and *mis*L) of *S. enterica* in North America, Africa, Oceania, Asia, Europe, and South America of each habitat. The colors represent habitats. Each bar represents the abundance of a specific virulence gene, with segments representing habitats.

To obtain a comprehensive picture of *S. enterica* virulence genes, we investigated the distribution of both *S. enterica* strains and phages that carry these virulence genes. A total of 466,136 *S. enterica* genomes were obtained from EnteroBase, of which 122,680 genomes (26.3%) had habitat information and belonged to 548 serotypes across 66 countries (Table S1). The *S. enterica* strains were primarily distributed across the Americas, spanning from 60° W to 150° W, followed by Europe (0°–30° E) and Asia (90° E–120° E) (Figure 1A). North America had the highest abundance of *S. enterica* (99,492 strains), followed by Asia (7344 strains) and Europe (7244 strains). Specifically, the countries with the highest abundance of *S. enterica* were the US (86,861 strains) and Canada (10,570 strains), followed by the UK (4451 strains), China (3925 strains), and Chile (2341 strains). The main habitats of *S. enterica* included food (39%), human and animal feces (32%), animals (15.2%), water (7.7%), sediments (0.8%), and soil (0.4%) (Figure 1B,C).

**FIGURE 2:**
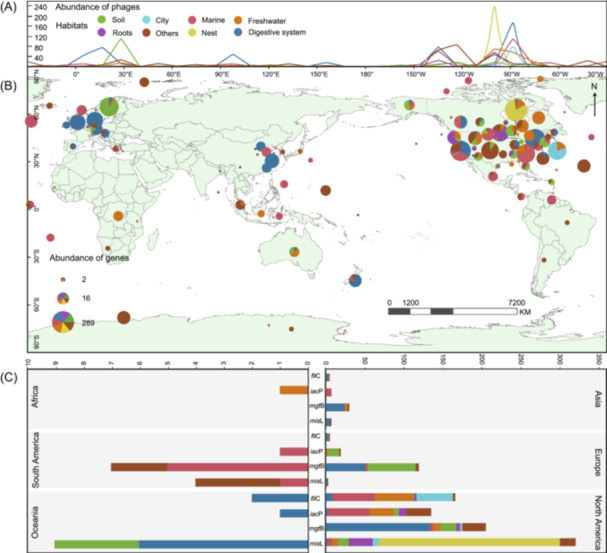
Global distribution of phages encoding *S. enterica* virulence genes. (A) The pattern of global variation in phage abundance with longitude. The *x*‐axis represents longitude and corresponds exactly to the longitude of the map. The *y*‐axis represents the abundance of phages carrying virulence genes. The colors represent habitats. (B) Global distribution of phages encoding *S. enterica* virulence genes in different habitats. The size of the pie chart represents the abundance of phages. And the colors represent habitats. (C) Abundance of the four genes of interest (*fli*C, *iac*P, *mgt*B, and *mis*L) in the phages in North America, Africa, Oceania, Asia, Europe, and South America of each habitat. The colors represent habitats. Each bar represents the abundance of a specific virulence gene, with segments representing habitats.

Similarly, we investigated the geographic distribution of phages carrying *S. enterica* virulence genes. A total of 5178 *S. enterica* virulence genes (*fli*C, *iac*P, *mgt*B/C, *mis*L, *sit*C, etc.) encoded‐phages were obtained from the IMG/VR database, which were primarily identified as *Caudoviricetes* (Tables S2 and S3). Of these phages, 45.73% of the phages were temperate, 33.49% were virulent, and the remaining 20.77% had uncertain lifestyles (Table S4). North America (60° W−150° W), Europe (0°−30° E), and Asia (90° E−120° E) were the top three regions reporting the highest abundance of phages carrying *S. enterica* virulence genes (Figure 2A). Specifically, the countries with the highest abundance of phages carrying *S. enterica* virulence genes were the US (1401), followed by Sweden (126), Canada (92), Denmark (84), and China (80). These phages were primarily found in six habitats, including mammalian digestive system (457), marine environments (362), nest (236), freshwater (199), soil (175), and roots (81) (Figure 2B,C). Additionally, we explored the kilopercentage ratios of uncultivated virus genomes (UViGs) carrying *S. enterica* virulence genes in the IMG/VR database across all habitats. Notably, the human digestive system had a relatively high prevalence of phages carrying *S. enterica* virulence genes, at 0.56‰ (Figure S1 and Table S5). Overall, the results demonstrate the widespread distribution of virulence genes in both *S. enterica* and its phages across diverse habitats worldwide, highlighting the significant threats they pose to global human health.

### Phage‐mediated horizontal transfer of virulence genes

**FIGURE 3:**
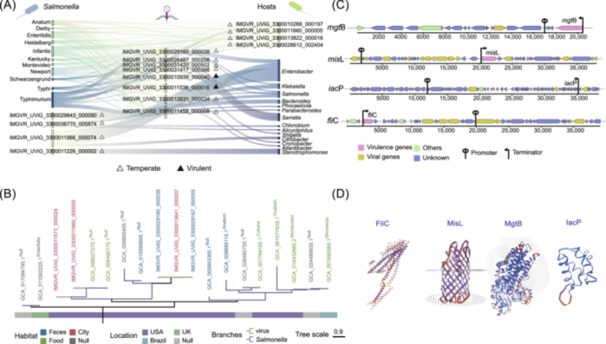
Phage host prediction and phylogenetic analysis of virulence genes. (A) Linkage between phages carrying virulence genes with *S. enterica,* as well as other bacteria. Left: the *S. enterica* serotypes that can match to phages carrying virulence genes, and phages match to host bacteria. Middle: the phages that can match to host. Right: bacterial hosts at the genus level, and phages only match to *S. enterica*. (B) Phylogenetic analysis of virulence gene *fli*C encoded by phages and *S. enterica*. The branch length represents the tree scale. The green branch represented a phage‐carried *fli*C, and the blue branch represented a *Salmonella*‐carried *fli*C. The color of the leaves name represents the habitat where the *fli*C is located. The bar next to the phylogenetic tree annotates the geographic location where *fli*C is located. (C) Genomic structures of phage‐encoded virulence genes. Pink represents virulence genes, yellow represents viral genes assigned V flags by DRAMV, green represents other types of genes identified, and blue represents genes with unidentified functions. (D) Predicted quaternary structures of phage‐encoded virulence genes (*fli*C, *iac*P, *mgt*B, and *mis*L).

To explore the evolutionary history of phages carrying virulence genes and gene exchange between phages and bacteria, we analyzed phage‐bacteria interactions, the phylogenies of virulence genes encoded by both phages and *S. enterica*, and the predicted protein quaternary structures of phage‐encoded virulence genes. Determining the phage‐host linkage is essential for confirming the occurrence of phage‐mediated HGT. A total of 52 phages were linked to 89,871 host *S. enterica* strains, covering a wide range of *S. enterica* serotypes, for example, Typhimurium, Infantis, Enteritidis, Newport, Heidelberg, Anatum, Derby, Schwarzengrund, Montevideo, and Typhi (Figure 3A, Tables S6 and S7). This suggests a high likelihood of HGT occurring between phages carrying virulence genes and several *S. enterica* serotypes. Notably, *S*. Typhimurium and *S*. Infantis were the most frequently targeted serotypes by phages, with 9204 *S*. Typhimurium and 7746 *S*. Infantis strains linked to 42 and 39 phages, respectively. Together, phages carrying virulence genes can infect a wide range of *S. enterica* serotypes, highlighting their crucial role in facilitating the transfer of virulence genes across different *S. enterica* serotypes.

To obtain more direct evidence of HGT between phages and *S. enterica*, we compared the affinities of the virulence genes encoded by *S. enterica* and phages. Phylogenetic tree showed that the virulence genes carried by phages were genetically close to those carried by *S. enterica* (Figures 3B, S2 and S3). For example, the branches of phage‐encoded *fli*C (IMGVR_UViG_3300011660_000005 and IMGVR_UViG_3300011573_000024) and the neighboring branches of *fli*C encoded by *S. enterica* strains (GCA_006027275.1 and GCA_006490175.1) shared the same ancestor. Moreover, the identity and coverage of *fli*C between *S. enterica* strains (GCA_006027275.1 and GCA_006490175.1) and phage (IMGVR_UViG_3300011660_000005) were equal to 100%. The identity of *fli*C between *S. enterica* strains (GCA_006027275.1 and GCA_006490175.1) and phage (IMGVR_UViG_3300011573_000024) was equal to 97.76% with 100% coverage. Phylogenetic analysis of both *mgt*B and *mis*L also revealed highly similar virulence gene segments (Table S8, Figures S2 and S3). The above *S. enterica* and phages carried homologous virulence genes with high identity values, indicating that HGT of virulence genes had occurred between phage and *S. enterica*.

To investigate the expression capacity of the virulence genes, we analyzed the locations of promoters and terminators and predicted the quaternary structure of *fli*C, *iac*P, *mgt*B, and *mis*L, all of which play important roles in *S. enterica* survival and pathogenicity. These genes were found to be positioned downstream of the promoter and upstream of the terminator (Figure 3C,D and Table S9). Based on SWISS‐MODEL analysis, the Global Model Quality Estimation (GMQE) indices for *fli*C, *iac*P, *mgt*B, and *mis*L were 0.80, 0.81, 0.84, and 0.88, respectively, indicating a high quality of prediction for these virulence genes carried by phages.

### The effect of *S. enterica* regulator *csr*A on phage‐mediated HGT

**FIGURE 4:**
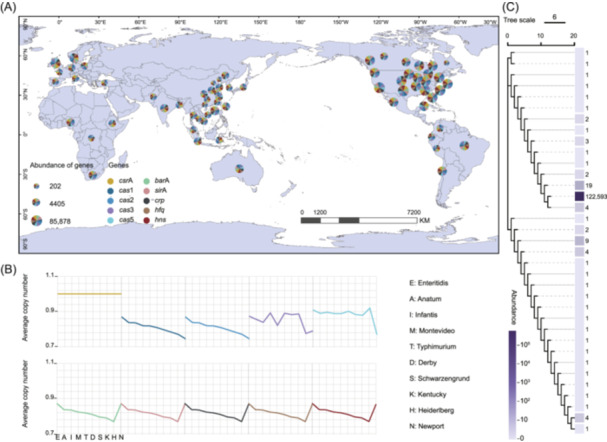
Global distribution of regulators in *S. enterica*. (A) Global distribution of virulence regulators encoded by *S. enterica*. Different colors represent different regulatory genes. And the size of the pie chart represents the abundance of genes. (B) The average copy number of regulatory genes changes with 10 serotypes from left to right: *S*. Enteritidis, *S*. Anatum, *S*. Infantis, *S*. Montevideo, *S*. Typhimurium, *S*. Derby, *S*. Schwarzengrund, *S*. Kentucky, *S*. Heidelberg, and *S*. Newport. (C) Phylogenetic tree of *csr*A. The branch length represents the tree scale. The bar and number next to it annotates the count of *csr*A in each branch.

Although virulence genes carried by viral vectors can be transmitted to different *S. enterica* serotypes, *S. enterica* has evolved different regulatory factors to mediate HGT and gene expression processes. In this study, we selected 10 regulatory factors in *S. enterica* based on their widespread distribution and universal regulatory functions. These factors, which were summarized from previous research, include *csr*A, *hns*, *hfq*, *crp*, *bar*A, *sir*A, *cas*1, *cas*2, *cas*3, and *cas*5. The regulatory mechanisms of these factors are illustrated in Figure S4. Regulatory genes in the *S. enterica* genomes were identified through BLAST analysis. The most frequently detected regulator was *csr*A, with a total of 122,681 occurrences, followed by *hns*, *hfq*, *crp*, and others, across the 122,680 *S. enterica* genomes. Similar to the distribution of virulence gene‐carrying *S. enterica*, the 10 regulator factors were also most extensively found in North America, followed by Asia and Europe. Their primary habitats included food, feces, and water (Figure 4A).

The average copy number of each regulator was also calculated for the top 10 *S. enterica* serotypes, including Enteritidis, Anatum, Infantis, Montevideo, Typhimurium, Derby, Schwarzengrund, Kentucky, Heidelberg, and Newport. The average copy number of the 10 regulators was generally high across the top 10 *S. enterica* serotypes, ranging from 0.7440 to 1.0000. The highest average copy number of the regulators was observed in *S*. Enteritidis, such as *csr*A (1.0000), *bar*A (0.8720), *sir*A (0.8721), *cas*1 (0.8696), *cas*2 (0.8696), *crp* (0.8722), *hfq* (0.8722), and *hns* (0.8722). Notably, the average copy number of *csr*A was the highest, which was almost 100% in all these serotypes (Figure 4B). In addition, 99.94% of the *csr*A had identical amino acid sequences (Figure 4C), suggesting the gene being highly conservative in *S. enterica*. Together, we found that *csr*A was the most widespread regulatory gene in *S. enterica* and was highly conserved across different serotypes. Therefore, *csr*A was selected for further investigation to validate its role in phage‐mediated HGT processes in *S. enterica*.

**FIGURE 5:**
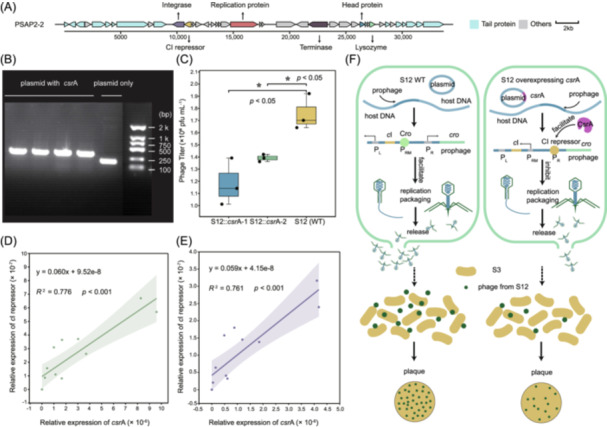
Experiments to validate the effect of *csr*A on phage cyclization and *c*I repressor. (A) The complete genomic structure of the prophage PSAP2‐2. Specific genes are indicated with arrows, with cyan for tail protein genes and gray for other genes. (B) Strains with *E. coli* plasmid PHB20TG carrying *csr*A and strain with only plasmid PHB20TG as a control were successfully constructed. (C) Phage titers of strains overexpressing *csr*A (S12::*csr*A‐1 and S12::*csr*A‐2) and wild‐type strain (WT) after induction of prophage expression. *p* < 0.05 indicated significant difference between strains overexpressing *csr*A and WT strain. The relationship between the expression of the *c*I repressor and *csr*A at a dilution gradient of 1:16 (D) and 1:32 (E), respectively. *p* < 0.001 indicated a significant correlation between the expression of *csr*A and that of the *c*I repressor. (F) Schematic representation of the main steps and results of the experiment. The green oval boxes on the left and right represent *S*. Typhimurium S12 carrying an empty plasmid and a plasmid containing the *csr*A gene via electroporation method, respectively. The prophage PSAP2‐2 inserted in the host DNA is determined by the expression of its own CI repressor to enter the lysis cycle. Upon induction, the CI repressor is cleaved, the *cro* gene‐expressed protein binds the RM promoter (*P*_RM_), represses the expression of *c*I, opens the left and right promoters (*P*_L_ and *P*_R_), and the prophage enters the lysis cycle. However, the overexpression of *csr*A promotes the expression of *c*I repressor, which activates the RM promoter (*P*_RM_), so that the phage tends to maintain the lysogenic cycle and reduces the release of phages. Released phages were infiltrated with *S*. Typhimurium S3, and the influence of *csr*A on prophage cyclization and release was quantified based on the count of phage spots on the petri dishes.

To validate the role of *csr*A in phage‐mediated HGT, we performed experiments to explore the effect of *csr*A on prophage cyclization and the *c*I repressor in *S*. Typhimurium strain S12. The prophage PSAP2‐2‐carrying S12 was selected as the representative *S. enterica* strain, and the *c*I repressor in the PSAP2‐2 genome was shown to mediate the phage life cycle (Figure 5A). Plasmids carrying *csr*A were introduced into *S*. Typhimurium strains via electroporation (Figure 5B and Table S10). The ability of the S12::*csr*A‐1 and S12::*csr*A‐2 with WT S12 to induce the cyclization and release of the prophage PASAP2‐2 was compared. We found that the average titer of phages released by S12::*csr*A‐1, S12::*csr*A‐2, and WT were 1.18 × 10^6^, 1.39 × 10^6^, and 1.75 × 10^6^ pfu mL^−1^, respectively. Notably, the phages released by S12::*csr*A‐1 and S12::*csr*A‐2 were significantly fewer than those released by the WT strain (*p* < 0.05; Figure 5C), indicating that *csr*A inhibited the cyclization and release of the prophage PSAP2‐2.

To investigate the regulatory mechanism of host gene *csr*A on prophage cyclization and release, we further determined the correlation between the expression of *csr*A and *c*I, which inhibits prophage cyclization by quantitative PCR (qPCR). We measured the corresponding bacterial optical density at 600 nm (OD_600_) at 2, 3, 4, 5, 7, and 9 h (Figure S5) and calculated the relative expression of *csr*A and *c*I based on their Ct values. A strong positive correlation was observed between the expression of *csr*A and *c*I at dilution of 1:16 (*R* ^2^ = 0.776, *p* < 0.001; Figures 5D) and 1:32 (*R* ^2^ = 0.761, *p* < 0.001; Figure 5E), which indicated that *csr*A inhibited prophage cyclization by promoting *c*I expression. In conclusion, the *S. enterica* regulatory gene *csr*A was found to regulate the phage life cycle, inhibit phage infection of *S. enterica*, and reduce the likelihood of horizontal transfer of *S. enterica* virulence genes (Figure 5F).

## DISCUSSION

### Virulence genes of *S. enterica* are widely found in phages

*S. enterica* infection represents a major threat to human health and has resulted in considerable economic losses worldwide. However, most existing studies have primarily focused on regional distribution and antibiotic resistance, especially β‐lactamase genes of *S. enterica* [25, 26]. These studies do not provide a comprehensive overview of the global distribution patterns, habitats, and virulence genes carried by *S. enterica*. This study is the first to investigate the global geographic distribution of *S. enterica*. In this study, 122,680 *S. enterica* were mainly observed in food, feces, and animals in North America, Europe, and Asia (Figure 1). Consistent with previous regional studies, we found that the average abundance of *S. enterica* virulence genes and Salmonellosis outbreaks are associated with gross domestic product and population density [27, 28]. The global distribution patterns of *S. enterica* can help raise awareness of food safety and public health concerns in countries with a higher risk of *Salmonella* outbreaks.

As phages can encode and facilitate the transmission of *S. enterica* virulence genes through HGT, we also investigated the global distribution of phages carrying *S. enterica* virulence genes. We identified 1844 phages that carry *S. enterica* virulence genes, which were predominantly found in the digestive system, marine environments, and nests across North America, Europe, and Asia (Figure 2), demonstrating that phages are important carriers of virulence genes in addition to *S. enterica*. Moreover, the digestive system was the habitat with the highest abundance and proportion of phages carrying *S. enterica* virulence genes. *S. enterica* was also commonly detected in food, feces, and animal bodies, which aligns well with the habitats of the phages. Notably, *fli*C, *mgt*B, *mis*L, and *iac*P were detected for the first time in phage genomes and identified as phage‐encoded auxiliary metabolic genes (AMGs) (Tables S11 and S12). These virulence genes, as phage‐encoded AMGs, not only alter the metabolism of host bacteria to enhance their virulence but also influence the spread of these genes within the bacterial community through phage‐mediated HGT. Together, this study offers a more comprehensive understanding of *S. enterica* virulence gene‐encoded phages and addresses several gaps in previous research.

### Counteracting effects of *S. enterica* and phages on horizontal transfer of virulence genes

The study of phage‐mediated horizontal transfer of *S. enterica* virulence genes is crucial for understanding how phages influence the pathogenicity of this bacterium. Based on phylogenetic analysis, we found that virulence genes encoded by both *S. enterica* and phages shared common ancestors (Figure 3B) with high identity and coverage, suggesting that HGT between phages and *S. enterica* has occurred historically. Based on the methods for identifying HGT among bacteria described in previous studies, horizontally transferred fragments should be aligned using BLAST, with an identity of 100% and a length greater than 500 bp [29]. Specifically, the amino acid residues of the virulence gene *fli*C encoded by phage IMGVR_UViG_3300011660_000005 matched exactly with those carried by *S. enterica* strains (GCA_006490175.1 and GCA_006027275.1), which aligns with the method described above for identifying HGT. We excluded the possibility that the bacterial sequences carrying *fli*C might be prophages, further confirming that phage IMGVR_UViG_3300011660_000005 is responsible for transferring *fli*C from *S. enterica*. Moreover, it was predicted that the phage‐encoded *fli*C has a structurally intact protein and performs its function, as determined by SWISS‐MODEL (GMQE = 0.81). However, for most *S. enterica* and phages, the virulence genes they carried were not 100% identical to each other, suggesting that base mutations may have occurred. It is likely that phages initially served as vectors to package bacterial virulence genes and subsequently underwent rapid sequence drift during their lifecycle [30]. This indicates that phages play a major role in the transfer of virulence genes. Together, our work provides new evidence of the flow and evolution of virulence genes within microbial communities by investigating the phylogenetic relationships and sequence identity of virulence genes encoded by both phages and *S. enterica*.

Although the virulence genes encoded by phages are promising in assisting host bacterial virulence‐related metabolism and pathogenicity, bacteria also possess their own regulatory genes to control the transfer of exogenous genes, including phage‐encoded genes [31, 32]. The 10 *S. enterica* regulator genes examined in this study were widely distributed worldwide, which were detected in 70.6% of *S. enterica* (Table S1). Among these, the *csr*A gene was the most extensively detected, highly conserved, and present across various serotypes (Figure 4), suggesting its significant role in *S. enterica*. This was supported by previous studies, which reported that *csr*A regulated the expression of 1025 bacterial genes associated with carbon metabolism and transport, iron metabolism, and cell envelope [33, 34, 35]. Notably, to the best of our knowledge, this is the first work that reports the linkage between phage‐mediated transduction and the bacterial regulator gene *csr*A. As shown in Figure 5, the titer of phages released by the strain overexpressing *csr*A was significantly lower than that of phages released by wild‐type strains (*p* < 0.05), and *csr*A expression significantly promoted *c*I expression of prophages (*p* < 0.001). The inhibitory effect of *csr*A on phage entry into the lysis cycle is consistent with previous studies that reported the overall negative effect of *csr*A on gene expression [33, 36]. Together, we identified the importance of the global regulator gene *csr*A in *S. enterica* and demonstrated for the first time that *csr*A inhibits prophage release. The inhibition suggests that *csr*A may reduce phage infection in bacteria, which could indirectly mitigate the impact of phage‐mediated horizontal transfer of virulence genes, thereby influencing the evolution of bacterial communities.

### Phage‐mediated horizontal transfer of *S. enterica* virulence genes requires further attention

Considering both phage‐encoded virulence genes and regulators, it is crucial to remain vigilant about phage‐mediated transmission of *S. enterica* virulence factors. While this large‐scale, data‐driven investigation provides valuable insights, it is important to acknowledge that the stringent quality filtering employed, combined with inherent limitations stemming from incomplete metadata, may introduce unavoidable biases in the analytical outcomes [37, 38, 39]. These biases may lead to an overrepresentation of virulence genes in developed regions and an underestimation of the burden of enteric fever in low‐ and middle‐income countries [40, 41]. This disparity highlights substantial gaps in the monitoring and sequencing of *S. enterica* and phages, likely due to economic constraints [3], which hinder the ability to track and understand the global spread and evolution of virulence factors. Based on the current available data, 36 phages were identified as polyvalent phages, as they were linked to more than one bacterial species in this study (Figure 3A), indicating the significant potential of these phages for HGT. In addition, phage infection may enhance host bacterial virulence [42]. As validated in this study, the overall transmission of *S. enterica* virulence genes increased due to the phage‐mediated horizontal transfer and expression of virulence genes. Although the global regulator gene *csr*A reduced the frequency of HGT by inhibiting prophage cyclization and release, it did not completely block phage infection. Future research should focus more on the roles of virulence factors and regulators in phage applications. Phages act as both sources and sinks for bacterial genes, offering potential opportunities for disease treatment in the context of antibiotic abuse, while also complicating the challenge of treating bacterial diseases. To minimize the risk of HGT of virulence genes, therapeutic phages should either be free of these genes or engineered to remove any virulence genes they may carry. A more comprehensive investigation into the roles and mechanisms of regulators in HGT is critical for accurately assessing the risks associated with phage‐mediated transfer and the dissemination of host virulence genes. This study is therefore essential for improving the safety and effectiveness of phage therapy applications.

## CONCLUSION

In this study, we have uncovered a complex interplay between phages and *S. enterica*, a bacterial genus associated with a variety of diseases in humans and animals. Our research demonstrates that phages play a pivotal role in the horizontal transfer of virulence genes within *S. enterica*, thereby potentially enhancing its pathogenicity. However, this process is intricately balanced by regulatory genes within the host bacterium that suppress these transfers. Our findings show that virulence genes carried by both phages and *S. enterica* are not only widespread but also exhibit close genetic affinity, indicating a history of HGT events. This genetic proximity and shared ancestry underscore the evolutionary relationship between phages and their bacterial hosts, highlighting the dynamic nature of their coexistence. Despite the apparent propensity for HGT, our work also demonstrates that certain regulatory genes within *S. enterica*, such as *csr*A, act as natural defense mechanisms. The gene *csr*A, which is abundant in *S. enterica*, was found to impede the life cycles of prophage PSAP2‐2, by preventing its cyclization and subsequent release. This inhibition, in turn, reduces the likelihood of phage‐mediated HGT, thereby limiting the spread of virulence factors. In conclusion, these findings emphasize the crucial role of phages in driving gene exchange and evolution in *S. enterica*. They also suggest that the regulatory machinery of *S. enterica* is not only essential for modulating the bacterium's own physiology but also plays a significant role in controlling phage life cycles and shaping the dynamics of HGT processes. These insights into the interaction between phages and their hosts advance our understanding of the mechanisms underlying bacterial pathogenicity and may have significant implications for the development of innovative therapeutic strategies to treat bacterial infections.

## METHODS

### Global distribution of *S. enterica* and phages

To determine whether phages carry *S. enterica* virulence genes and to analyze the global distribution pattern of these genes in both phages and *S. enterica*, we collected as many reliable genomic datasets as possible and used them as a basis for mapping. The amino acid sequences of *S. enterica* target virulence genes (*fim*A, *fli*C, *iac*P, *mgt*B, *mgt*C, *mis*L, *pag*C, *spv*A/B/C/R, *sse*I, *ara*B, *bcf*C, and *sit*C) and regulatory genes (*csr*A, *hns*, *hfq*, *crp*, *bar*A, *sir*A, *cas*1, *cas*2, *cas*3, and *cas*5) were downloaded from the KEGG database, while 466,136 *S. enterica* genomes were downloaded from EnteroBase (https://enterobase.warwick.ac.uk/, accessed on 29 May 2023) [43]. The region encoding these target genes in *S. enterica* was identified through BLAST (v2.13.0+) and filtered using the following parameters: *E*‐value < 0.0001, identity >70%, and coverage >70%. After filtering the fields “Country” and “Isolation Source,” 122,680 *S. enterica* genomes remained. Similarly, the target amino acid sequences were blasted against the IMG/VR v4 database, which contains over 15,000,000 viral genomes and genome fragments (https://img.jgi.doe.gov/cgi-bin/vr/main.cgi) [44]. After removing redundant matches, 1839 phages encoding *S. enterica* virulence genes were identified (*E*‐value < 0.0001, bit score > 40, and identity > 20%). Finally, *S. enterica* and phages were spatially visualized at the national level using ArcGIS 10.7 (ESRI) on a global scale, based on geographic location and habitat. The information on *S. enterica* and phages carrying *S. enterica* virulence genes is provided in Tables S1 and S2.

To investigate the proportion of uncultivated viral genomes UViGs encoding *S. enterica* virulence genes in each habitat, we calculated the ratios of these UViGs in this study to the total number of UViGs in the IMG/VR database. Habitats with three or fewer sampling sites or projects were excluded from this study to minimize the bias of single sample on the results. The abundance of UViGs in each habitat, both in this study and the IMG/VR database, is shown in Table S3.

### Taxonomic assignment and functional annotation of phages

To classify the phages, the obtained phage genomes were scanned using HMMER (v3.1b2), aligning protein sequences with HMM models corresponding to specific phage taxon from the ViPhOG database [45] (Table S3). The hit with the highest score for each sequence was selected as the best assignment. The data set was further filtered and annotated using DRAMV [46]. AMGs were identified by combining DRAMV (v1.2.0) with VIBRANT (v1.2.0) using default parameters. The potential functions of the identified genes, including AMGs, were further validated through protein structure prediction. The SWISS‐MODEL server (https://swissmodel.expasy.org/, accessed on 11 December 2021) was used to predict the quaternary structure of each protein with GMQE score > 0.5. The BPROM server (http://www.softberry.com/, accessed on January 2, 2022) and the FindTerm server (http://www.softberry.com/, accessed on January 2, 2022) were then used to predict the promoter (threshold: 0.20) and terminator of phage‐encoded virulence genes, respectively.

### Phylogenetic analysis, phage lifestyle, and putative host prediction

Amino acid sequences corresponding to virulence genes or regulatory genes encoded by phages and *S. enterica* were aligned using MUSCLE (v5.3) [47] and then trimmed with parameter 0.5. Trees were constructed using either IQ‐TREE (v1.5.5) [48] or MEGA11 [49]. Bootstrapping with 1000 replications was performed to assess the statistical robustness of the clustering. Phage lifestyles, including virulent and temperate, were predicted using a new model based on BERT, PhaTYP (http://phage.ee.cityu.edu.hk/phatyp, accessed on June 20, 2022 [50]; Table S4). The nucleotide sequences were inputted into the model and assigned probabilities for each lifestyle class. Two prognostic computational approaches genome (homology match and CRISPR match) were used to predict putative phage‐host linkages. The genomes of *S. enterica* were used to search for CRISPR spacers using CRISPRFinder (v4.3.2) [51]. Phage genomic signatures in *S. enterica* were identified via BLAST with the following parameters: *E*‐value < 0.0001, bit score ≥ 50, identity ≥ 70%, and matching length ≥ 2500 bp [52] (Tables S6 and S7). Moreover, phages linked to multiple bacterial genera were classified as polyvalent phages, meaning they can infect a wide range of hosts.

### Bench trials to validate the role of regulators on HGT of *S. enterica* virulence genes

We conducted an experiment to investigate the role of *csr*A in mediating phage‐mediated horizontal transfer processes. The cyclization and release of the prophage PSAP2‐2 were compared between wild‐type *S. enterica* subsp. *enterica* serotype Typhimurium (*S*. Typhimurium) strain S12 (WT) and *S*. Typhimurium strain S12 overexpressing *csr*A (i.e., S12::*csr*A‐1 and S12::*csr*A‐2). If the release of prophages decreased in strains overexpressing *csr*A, the probability of HGT would be lower in these strains compared to the control. The phage titer (pfu mL^−1^) of S12::*csr*A‐1, S12::*csr*A‐2, and WT strains was determined after induction of prophage expression. The expression of *csr*A and the *c*I repressor was measured by qPCR.

Construction of strains overexpressing *csr*A. The primers used in this study are listed in Table S10. The *E. coli* plasmid PHB20TG contained recombinant enzymes that can be expressed by l‐arabinose, along with a gentamicin resistance gene used as a screening marker. The desired gene sequence was amplified by PCR using primers S77 and S78. The PCR product was then cloned into the digested plasmid vector. DNA ligase was used to ligate *csr*A into the excised vector. Strain S12 was cultured to the logarithmic phase to prepare competent cells. A volume of 10 µL of the extracted plasmid was electroporated into the S12 cells, which were then cultured at 37°C. Two monoclonal colonies were randomly selected for purification on plates containing 15 µg mL^−1^ gentamicin and incubated at 37°C. Strains S12::*csr*A‐1 and S12::*csr*A‐2 were constructed from the two clones, respectively. The control strain was WT carrying the plasmid PHB20TG without *csr*A. The mutant was further confirmed by DNA sequencing of the PCR products.

Determination of phage titer. Strains overexpressing *csr*A and the WT strain were incubated overnight at 37°C, and then transferred to 3 mL of LB containing 3 µL of 15 µg mL^−1^ gentamicin and 60 µL of 20% arabinose. The strains were incubated at 37°C with shaking for 6 h, and the OD_600_ was determined. Bacterial suspensions were centrifuged at 12,000 *g* and 4°C for 5 min. The supernatant was collected and diluted to 10^−2^, 10^−3^, and 10^−4^ of the original concentration. A volume of 10 µL from each diluted suspension was taken, along with 200 µL of S3, incubated at 37°C, and the plaque‐forming units (pfu) were counted. The experiment was set up with three parallel control groups. Phage titer was calculated following the equation listed below

(1) $$P{\text{hage}\;\text{titer}(\text{pfu mL}}^{-1})=\frac{\text{Plaque}\;\text{forming}\;\text{unit}\;(\text{pfu})\times \text{Dilution}}{\text{Volume}\;(\text{mL})},$$

qPCR and analysis of *csr*A and *c*I repressor. The S12 strain was cultured in 15 mL of LB medium and incubated at 37°C with shaking for 2, 3, 4, 5, 7, and 9 h. At each time point, the OD_600_ was measured. Subsequently, 1 mL of bacterial culture was centrifuged at 12,000 *g* for 3 min at 4°C. RNA was extracted from the bacterial pellet using Trizol reagent and treated with DNase I to remove any residual DNA. The RNA concentration was then determined and standardized. The RNA was reverse‐transcribed into cDNA, which was diluted to 1:16 and 1:32. The experiment utilized two sets of primers, S85–86 and S87–88, for qPCR. The cycle threshold (*Ct*) values of *csr*A and the *c*I repressor were obtained. The correlation between the relative expression of *csr*A and *c*I repressor was analyzed using scatter plots and linear regression. The relative expression was calculated following the equation listed below,

(2) $$R\text{elative}\;\text{expression}\;\text{of}\;\mathrm{gene}=2^{-Ct_{\mathrm{gene}}}.$$

### Statistical analysis

Differences in parameters were assessed for significance using *t*‐test, and variable correlations were analyzed using bivariate correlation in SPSS 26.0 (SPSS Inc). Data visualization was conducted using the website: https://www.chiplot.online/, and geographic distribution was visualized using ArcGIS 10.7 (Esri).

## AUTHOR CONTRIBUTIONS

**Tianjing She**: Writing—original draft; visualization; software; formal analysis; data curation; conceptualization; methodology. **Demeng Tan**: Conceptualization; methodology; software; supervision; resources; validation. **Jose Luis Balcazar**: Writing—review and editing; formal analysis; supervision. **Ville‐Petri Friman**: Writing—review and editing; formal analysis; supervision. **Danrui Wang**: Software; project administration; supervision; data curation. **Dong Zhu**: Methodology; formal analysis; supervision. **Mao Ye**: Conceptualization; writing—review and editing; methodology; formal analysis; project administration; supervision; resources. **Mingming Sun**: Supervision; resources; project administration; formal analysis; writing—review and editing; methodology; conceptualization; funding acquisition. **Shujian Yuan**: Investigation; conceptualization; methodology; data curation. **Feng Hu**: Funding acquisition; project administration; supervision; resources.

## CONFLICT OF INTEREST STATEMENT

The authors declare no conflicts of interest.

## ETHICS STATEMENT

No animals or humans were involved in this study.

## Supporting information


**Figure S1.** Percentage of uncultivated virus genomes (UViGs) carrying *Salmonella enterica* (*S. enterica*) virulence genes in each habitat.
**Figure S2.** Phylogenetic tree of *mgt*B encoded by *S. enterica* and phages.
**Figure S3.** Phylogenetic tree of *mis*L encoded by *S. enterica* and phages.
**Figure S4.** Regulatory mechanisms of the key regulators in *S. enterica*.
**Figure S5.** Bacterial optical density at 600 nm (OD_600_) when performing quantitative PCR (qPCR) experiments.

**Table S1.** Metadata of 122,680 filtered *Salmonella enterica* genomes.
**Table S2.** Metadata of 1839 filtered phage genomes.
**Table S3.** Phage taxonomy.
**Table S4.** Phage lifestyles.
**Table S5.** Proportion of UViGs in each habitat.
**Table S6.** Host predicted by CRISPR match.
**Table S7.** Host predicted by homology match.
**Table S8.** Alignment of virulence genes carried by *S. enterica* and phages via BLAST.
**Table S9.** Prediction of promoters and terminators.
**Table S10.** Sequences of genes and primers for qPCR experiments.
**Table S11.** Phage AMGs identified by DRAMV.
**Table S12.** Phage AMGs identified by VIBRANT.

## Data Availability

Data collected for the study are publicly available from the Figshare platform, and can be downloaded from https://doi.org/10.6084/m9.figshare.26493310, https://doi.org/10.6084/m9.figshare.26527759, and https://doi.org/10.6084/m9.figshare.26531836. The data and scripts used are saved in GitHub https://github.com/TianjingShe/Salmonella-enterica. Supplementary materials (figures, tables, graphical abstract, slides, videos, Chinese translated version, and update materials) may be found in the online DOI or iMeta Science http://www.imeta.science/.
