# Supplementary material for: Phage‐mediated horizontal transfer of Salmonella enterica virulence genes with regulatory feedback from the host
Source: Imeta. 2025 May 20;4(4):e70042. doi: 10.1002/imt2.70042 (PMC12371270; doi:10.1002/imt2.70042)
Supplement: Supplementary file 1 — Figure S1. Percentage of uncultivated virus genomes (UViGs) carrying Salmonella enterica (S. enterica) virulence genes in each habitat. Figure S2. Phylogenetic tree of mgtB encoded by S. enterica and phages. Figure S3. Phylogenetic tree of misL encoded by S. enterica and phages. Figure S4. Regulatory mechanisms of the key regulators in S. enterica. Figure S5. Bacterial optical density at 600 nm (OD600) when performing quantitative PCR (qPCR) experiments. [file IMT2-4-e70042-s001.docx]

**Supporting information to**

**Phage-mediated horizontal transfer of *Salmonella enterica* virulence genes with regulatory feedback from the host**

**Running title**: Phage-mediated horizontal transfer of *Salmonella* virulence genes

Tianjing She^1^, Demeng Tan^2^, Jose Luis Balcazar^3*^, Ville-Petri Friman^4^, Danrui Wang^1^, Dong Zhu^5^, Mao Ye^6,^ Mingming Sun^1*^, Shujian Yuan^1^, Feng Hu^1^

^1^Soil Ecology Lab, Jiangsu Collaborative Innovation Center for Solid Organic Waste Resource Utilization and Jiangsu Key Laboratory for Solid Organic Waste Utilization, Nanjing Agricultural University, Nanjing 210095, China

^2^Shanghai Public Health Clinical Center, Fudan University, Shanghai 201508, China

^3^Catalan Institute for Water Research ICRA, Girona 17003, Spain

^4^Department of Microbiology, University of Helsinki, Helsinki 00014, Finland

^5^Zhejiang Key Laboratory of Urban Environmental Processes and Pollution Control, Ningbo Urban Environment Observation and Research Station, Chinese Academy of Sciences, Ningbo 315800, China

^6^National Engineering Research Center for Soil Nutrient Management and Pollution Remediation, Institute of Soil Science, Chinese Academy of Sciences, Nanjing 210008, China

*Correspondence: jlbalcazar@icra.cat (Jose Luis Balcazar), [sunmingming@njau.edu.cn](mailto:sunmingming@njau.edu.cn) (Mingming Sun).


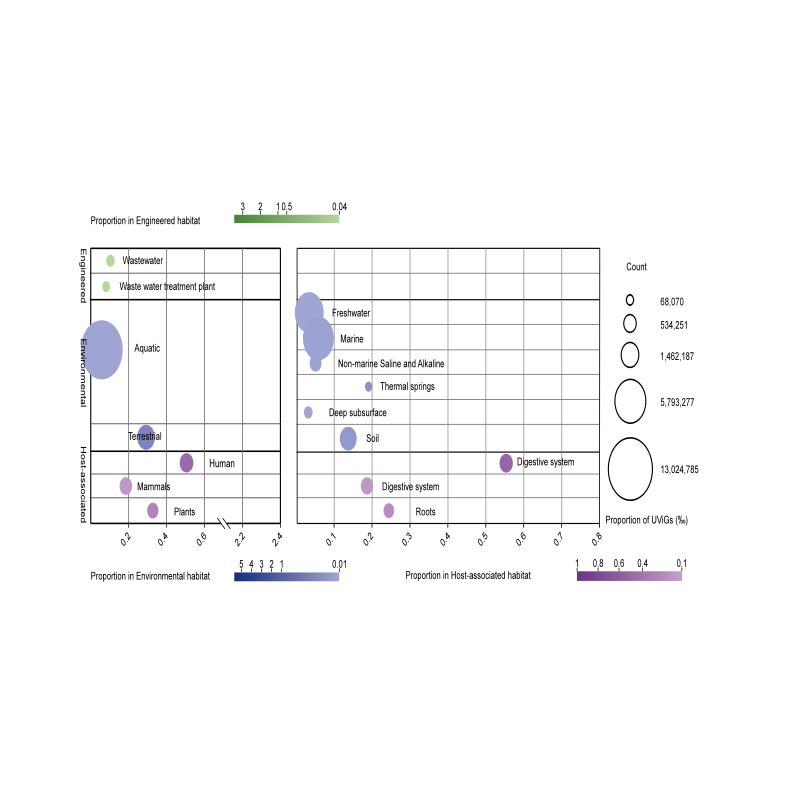


**Figure S1. Percentage of uncultivated virus genomes (UViGs) carrying *Salmonella enterica* (*S. enterica*) virulence genes in each habitat.** The circle size indicated the total number of phages in the Integrated Microbial Genomes and Virome (IMG/VR) database for the habitat. Green represents engineered habitats, blue represents natural habitats, and purple represents host-associated habitats. Secondary categorization of engineered, environmental, and host-associated habitats is shown in the left bubble chart, and third categorization of these habitats is further displayed in the right bubble chart. The darker the color, the higher the proportion of phages carrying *S. enterica* virulence genes in the habitat.

**
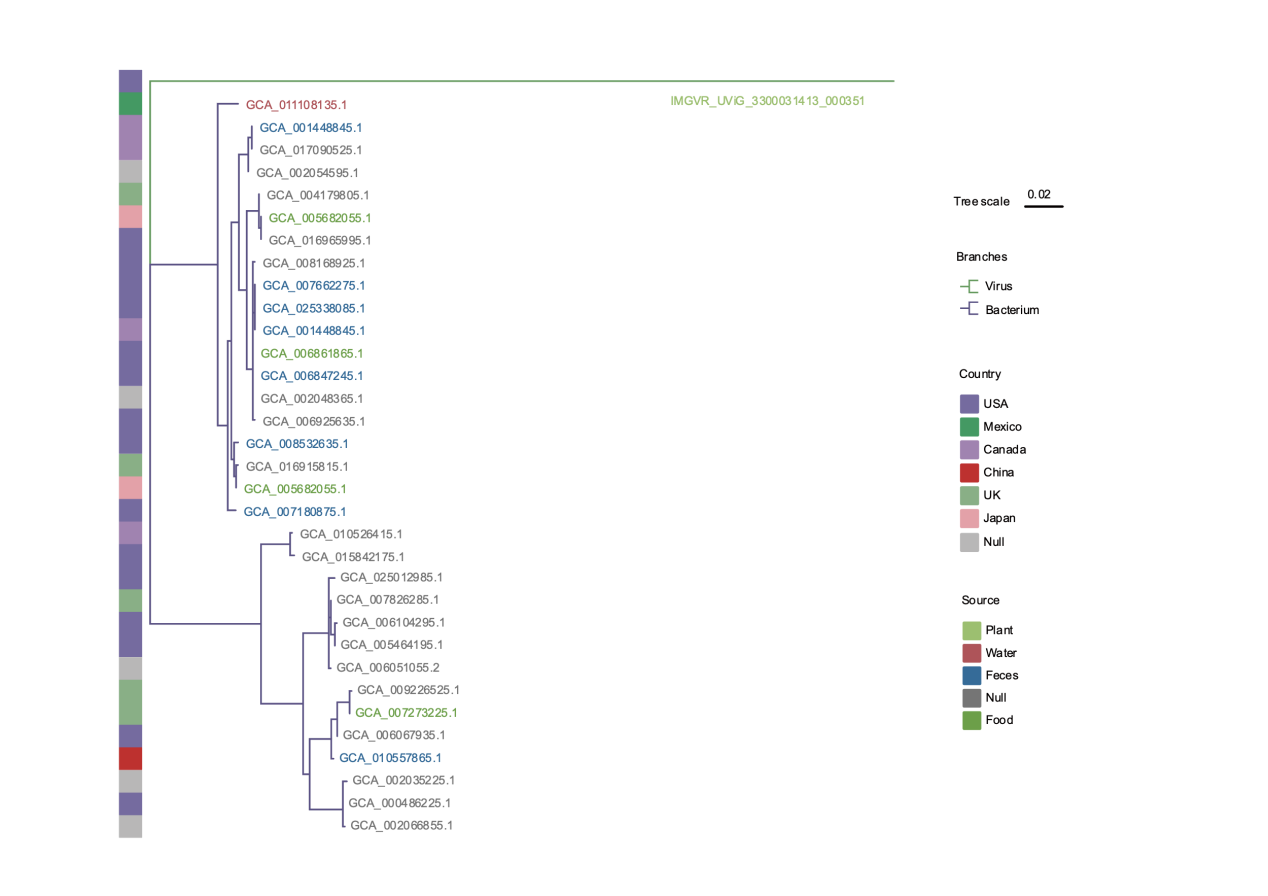
Figure S2. Phylogenetic tree of *S. enterica* and phages encoding *mgt*B.** The branch length represents the tree scale. The green branch represented a phage-carried *mgt*B, and the blue branch represented a *Salmonella*-carried *mgt*B. The color of the branch name represents the habitat where the *mgt*B is located. The bar next to the phylogenetic tree annotates the geographic location where *mgt*B is located.

**
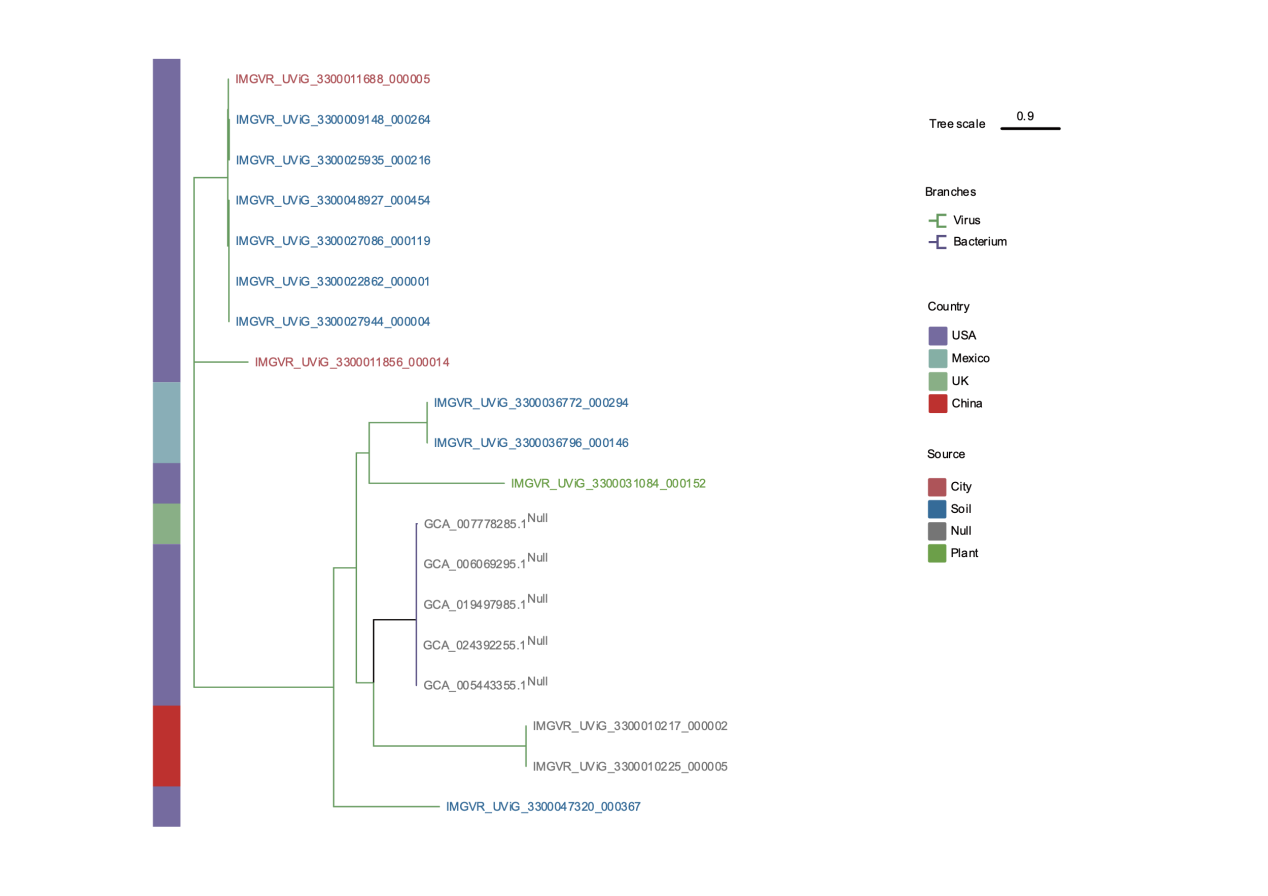
Figure S3. Phylogenetic tree of *S. enterica* and phages encoding *mis*L.** The branch length represents the tree scale. The green branch represented a phage-carried *mis*L, and the blue branch represented a *Salmonella*-carried *mis*L. The color of the branch name represents the habitat where the *mis*L is located. The bar next to the phylogenetic tree annotates the geographic location where *mis*L is located.

**
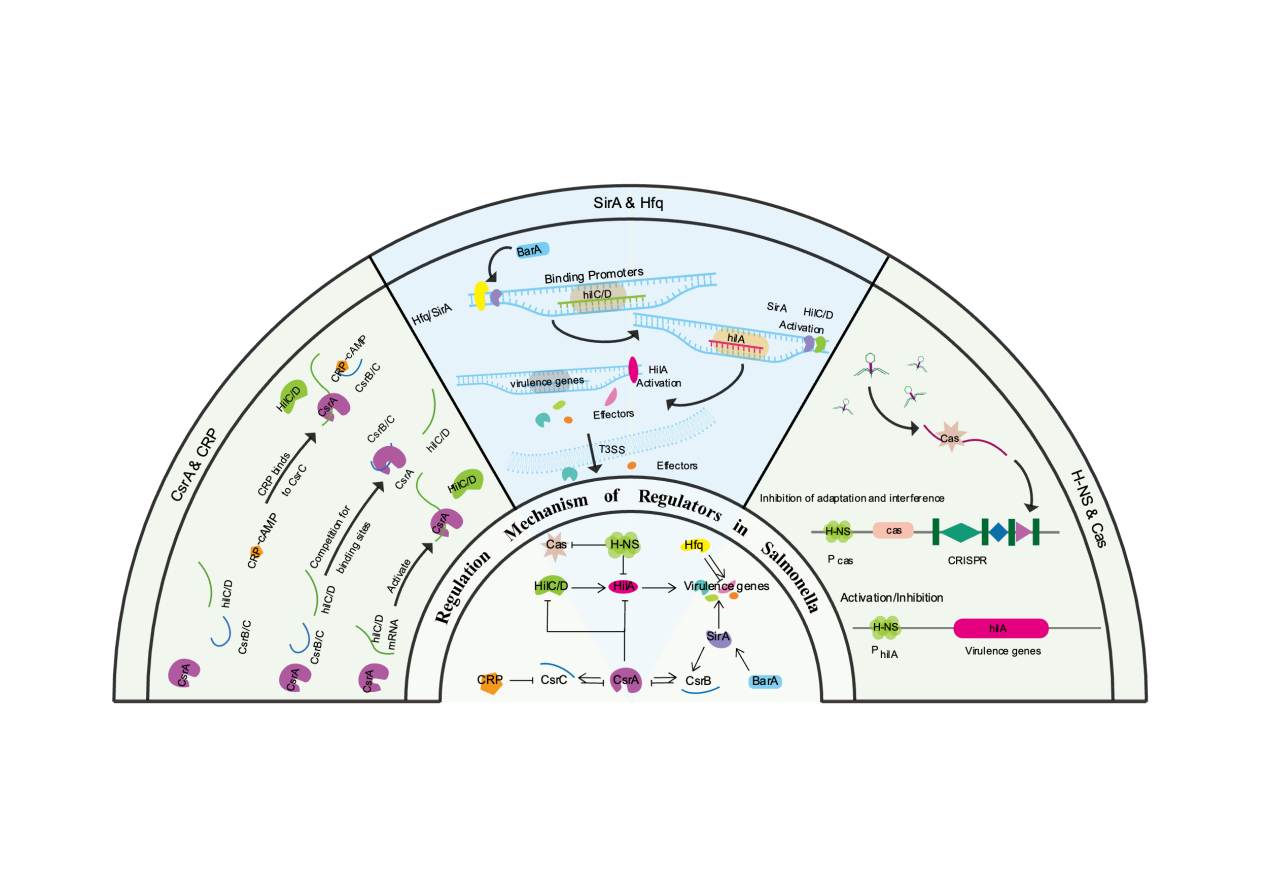
Figure S4. Regulatory mechanisms of the key regulators in *S. enterica*.** The regulatory mechanisms of the genes are delineated within the fan-shaped regions, while their integrative regulatory relationships are succinctly elucidated in the semicircular regions situated below. Specifically, The CsrA protein can promote the expression of HilC/D protein by binding to the *hil*C/D mRNA, thus affecting the expression of pathogenicity island genes. In contrast, whereas the *csr*B/C mRNAs repress HilC/D expression by competing for the binding site. Crp proteins, in turn, can regulate the csr system by binding to the *csr*C mRNA. BarA transduces the receptor signal to SirA, which acts similarly to Hfq, promoting *hil*A gene expression by binding to the promoter of the *hil*C/D gene, which further promotes virulence gene expression. The CRISPR-Cas system recognizes and defends against phage, while H-NS influences the CRISPR-Cas system by binding to the cas gene promoter. In addition, H-NS can also directly bind to other lower regulatory genes, thus affecting virulence and other vital activities of *S. enterica*.


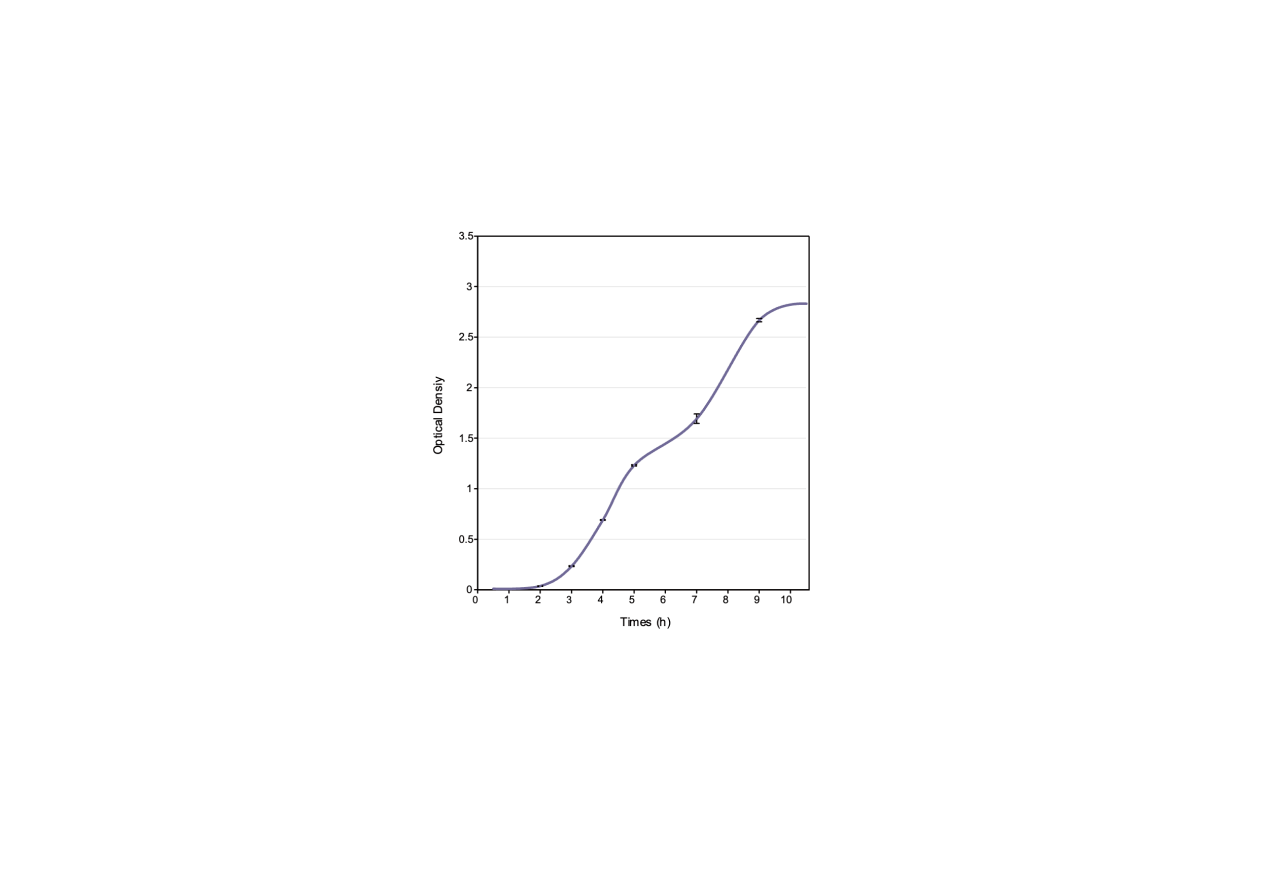


**Figure S5. Bacterial optical density at 600 nm (OD_600_) when performing quantitative PCR (qPCR) experiments.** OD values were determined when the bacteria were incubated at 37 ℃ for 2, 3, 4, 5, 7, and 9 h.
